# Defect-insensitive cylindrical surface lattice resonance array and its batch replication for enhanced immunoassay

**DOI:** 10.1038/s41378-024-00793-3

**Published:** 2024-11-13

**Authors:** Bin Zhou, Chao Hu, Haoyang Li, Xiangyi Ye, Baohua Wen, Zhangkai Zhou, Jingxuan Cai, Jianhua Zhou

**Affiliations:** 1 Key Laboratory of Sensing Technology and Biomedical Instruments of Guangdong Province, School of Biomedical Engineering, Shenzhen Campus of Sun Yat-sen University, 518107 Shenzhen, China; 2 School of Physics, State Key Laboratory of Optoelectronic Materials and Technologies, Sun Yat-sen University, 510275 Guangzhou, China

**Keywords:** Biosensors, Nanostructures

## Abstract

Surface lattice resonances (SLR) have been demonstrated to enhance the sensitivity and reduce the full width at half maximum (FWHM) of the plasmonic resonances. However, their widespread application in immunoassays has been hindered by limitations of high structural defect sensitivity and fabrication costs. Here, we design a novel three-layer cylindrical SLR array that exhibits high tolerance against structural defects, which would facilitate straightforward fabrication. By integrating metal evaporation and nanoimprint lithography, we demonstrate the replication of the SLR array with exceptional quality. Theoretical simulations indicate that the resonance dips of these arrays exhibit are not sensitive to various structural defects. The experimental results reveal that the FWHM of these arrays can be as low as 5.1 nm while maintaining robust resonance characteristics. Furthermore, we demonstrated the high spectral sensitivity of the SLR array, which enabled the detection of immunoglobulin G (IgG) at concentrations as low as 609 pg/mL. These findings emphasize the potential of the defect-insensitive SLR array as a highly scalable immunoassay platform with exceptional performance.

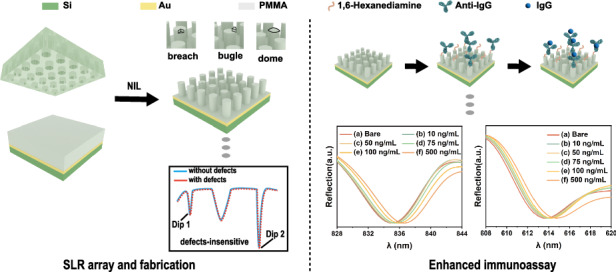

## Introduction

Plasmonic sensors exhibit significant potential in immunoassay and antigen-antibody interaction studies due to their high sensitivity, real-time operation capabilities, and label-free nature^1–6^. Currently, plasmonic sensors primarily detect absorbed target analyses and determine their concentrations through the shift of the resonance peak or dip (Δ*λ*), depending on the specific spectral measurement methods^1,5,7–9^. The sensitivity and limit of detection (LOD) of plasmonic sensors are mainly restricted by the FWHM of the resonance peak. A narrower FWHM enhances the ability to distinguish the peak shift. Therefore, reducing the FWHM of the resonance peak is an effective strategy to enhance the sensitivity and reduce the LOD of the plasmonic mode^5,10,11^. However, the broad FWHM of local surface plasmons (LSPs) and propagating surface plasmons (PSPs) in current plasmonic sensors limit their further application. Specifically, LSPs and PSPs often experience energy loss due to radiation damping and dynamic depolarization, resulting in a broad FWHM of the resonance peak^10^. For instance, the width of the LSPs resonance peak in Au nanostructures typically exceeds 80–100 nm, while that of PSPs is around 50 nm^5^, which limits the performance of plasmonic sensors for the detection of small amounts of molecules.

Electric field mode coupling is a crucial method to reduce energy loss in plasmonic sensors, thereby narrowing the FWHM of resonance peaks^10,12^. SLR is one of the most promising implementations^13,14^, achieved by arranging nanoparticles into ordered arrays to couple the lattice diffraction with localized surface plasmon resonance (LSPR), reducing energy loss and narrowing FWHM of resonance peaks^15–20^. Recent studies reported SLR structures with high *Q*-factors that exhibit high sensitivities in hydrogen gas sensing^7^. However, achieving narrow linewidth SLR peaks requires high-quality orderly arranged arrays^21^. The hybridization between the LSPR and the formation of the lattice modes was mainly controlled by the radiation pattern of the individual metal scatterers forming the arrays^22,23^. Better consistency of the array periodicity results in narrower SLR peaks^22^, which often rely on precision-demanding serial fabrication techniques like E-beam lithography (EBL)^7,17,24,25^. However, the high cost and vacuum-demanding requirements of the fabrication techniques limit the practical feasibility of the SLR array in sensing applications.

Nanoimprint lithography (NIL) is an excellent technique for large-scale production of nanostructures^26–28^ and can replicate features <10 nm over a large area with long-range order, which provides a new way for fabrication of the SLR array^27^. However, drawbacks of nanoimprint technology, such as polymer fracture in the thermal NIL, hinder its employment in the fabrication of high-quality nanostructures^29^. During the hot pressing, cooling, and releasing steps in the thermal NIL process, the applied pressure on the imprint resist structures after cooling below the glass transition temperature of resist materials induces a stress concentration at the corner of the resist structures. Differences in the thermal expansion coefficients of the mold and substrate materials induce lateral strain, which also concentrates at the corners of the structure, and results in fractures at the bottom of the structure during the demolding step^29^. Various forms of defects may also exist during the NIL process, such as breach, bugle, and dome^30^. In addition, during the NIL process, the template often suffers from permanent wearing after a few cycles of the NIL process^31,32^. Therefore, an optimized design of an array with high tolerance to structural defects can greatly improve the durability and application range of the nanoimprint template.

Here, we designed and fabricated a three-layer cylindrical SLR array using an improved NIL technique. The simulated SLR resonance peaks in the reflectance spectrum of this array exhibit high tolerance to the structural defects during the NIL process. This SLR array fabricated through an optimized NIL process demonstrates a narrow resonance dip with 5.1-nm FWHM and shows excellent batch-to-batch consistency. Theoretical and experimental investigations reveal a good refractive index sensitivity of the SLR array that is closely related to the structural period of the array. The potential capability of the SLR array in immunoassay is also presented with excellent sensing performance. We have successfully detected IgG target molecules with concentrations as low as 609 pg/mL, highlighting the potential of the SLR array as a highly sensitive platform for practical applications. This SLR array, with its high tolerance to structural defects and excellent immune detection performance, holds promise for future applications in immune detection analysis.

## Results and discussion

### Design of defect-insensitive SLR array

**Fig. 1:**
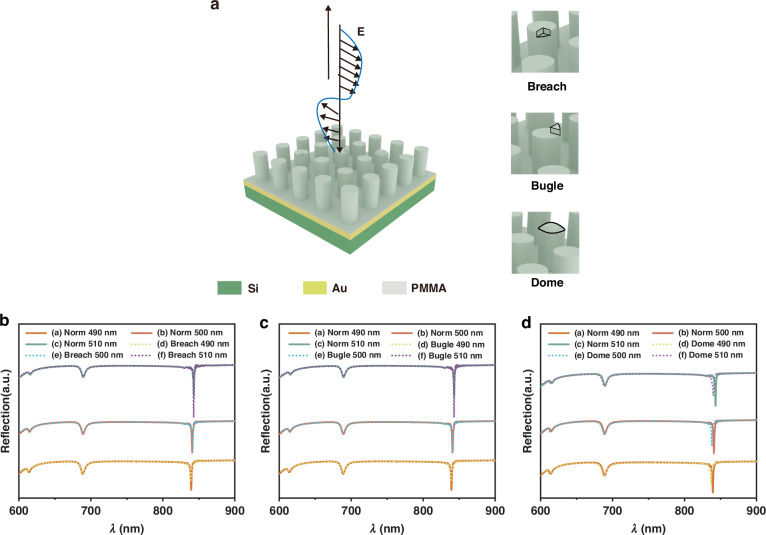
Simulation of reflectance spectra of SLR array with different types of structural defects: breach, bugle, and dome. **a** Schematic diagram showing the structure of the SLR array. **b** Reflectance spectra of the SLR array with defects of breach (fan-shaped pillar with a radius of 100 nm, height of 120 nm, and an angle of 90°) compared to the SLR array without defects. **c** Reflectance spectra of the SLR array with defects of a bugle (extra fan pillar with a radius of 100 nm, height of 120 nm, and an angle of 90°) compared to the SLR array without defects. **d** Reflectance spectra of the SLR array with defects of the dome (extra curved sphere with a radius of 100 nm, height of 30 nm, and spherical amplitudes of 25°) compared to the SLR array without defects

We designed a three-layer cylindrical SLR array, optimized its size parameters, and achieved its replication using the NIL technique (Fig. 1a). This array comprises a top layer of polymethyl methacrylate (PMMA) cylindrical structures, serving as a low refractive index dielectric layer. A middle layer of gold serves as the plasmon supply layer. A bottom layer of silicon substrate provides a support layer. The SLR array is designed to locate the SLR wavelength in the IR-visible region, and the reflectance spectra of the SLR array are studied afterward. Common defects such as breach, bugle, and dome structures that may be encountered during the NIL process are used to assess the designed structure’s tolerance to defects within the array. Finite-difference time-domain (FDTD) simulations were first performed to explore the effects of various parameters on the SLR structure, including diameter (*D*), height (*H*) of PMMA pillars, and the thickness (*T*) of the gold layer for optimization of the performance and investigation of the fabrication tolerance of the SLR array, the reflectance spectra as shown in Figs. 1 and S1, S2.

According to the SLR excitation wavelength formula^33^ :

$${\lambda }_{{{\rm {air}}}}=\frac{a}{m}[1\pm \sin \left(\theta \right)]$$

$${\lambda }_{{{\rm {sub}}}}=\frac{a}{m}[{n}_{{\rm {s}}}\pm \sin \left(\theta \right)]$$

*λ*_air_ and *λ*_sub_ are two resonance modes of SLR, and *a* is the period of the structure, and *m* is an integer, *θ* is the angle of incidence and *n*_s_ is the refractive index of the substrate. Based on the calculations, the first-order lattice resonance dip in the air is ~841 nm (dip 2) and the second-order lattice resonance dip on the dielectric surface is nearly 615 nm (dip 1). This directly proves that these two resonance dips are SLR dips.

Figure 1b demonstrates the typical SLR array without defects and with breach defects, respectively, which show a redshift and narrower dips of the resonance with an increasing pillar height H ranging from 490 to 510 nm. Similarly, we simulated the reflectance spectra of the SLR array with pillar diameters ranging from 480 to 530 nm (Fig. S1A). The results indicate that the diameter of the pillars does not significantly impact the resonance characteristics of the structure in both the wavelength and FWHM of the resonance dips. Figure 1c shows that the array with defects of bugle does not significantly broaden the FWHM, and the resonance dips also do not shift from the normal structures. To further investigate the defect tolerance of the SLR array, we studied the influence of defects of the dome on the spectral response by FDTD simulations, as shown in Fig. 1d. Compared to the SLR array without defects, the SLR array with defects of the dome has a minimal impact on the FWHM of the resonance dips in reflectance spectra, with an increasing pillar height *H* ranging from 490 to 510 nm. The results show that the FWHM of the resonance dips remain relatively unchanged with a slight blue shift within this range. The effects of other potential defects, such as breach of the bottom, curved bottom, and uneven bottom on the spectral response have been shown in Fig. S3. The peak positions at 615 and 841 nm remain unchanged, indicating that the array maintains a degree of tolerance to these structural variations.

Subsequently, we investigated the influence of the gold layer thickness on the SLR dips. Figure S1B indicates that the SLR dips are more dependent on the thickness of the gold layer. The FWHM of the SLR dips decreases with the thickness of the gold layer, as shown in Fig. S1C. The gold layer, which serves as the plasmon supply layer, affects the excitation of surface plasmon polaritons (SPP) thus affecting SLR. Furthermore, the efficiency of SPP excitation decreases when increasing film thickness due to the increase in the tunneling distance^34,35^, and the thickness can be altered by the material evaporation process over a large area. We decided to set both the diameter and height of the pillars to 500 nm. Besides, we selected an array configuration with a gold layer thickness of 100 nm and a period of 830 nm, for the simplicity of the fabrication in the subsequent process.

The designed SLR structure exhibits significant advantages, with excellent tolerance to structural defects, and can be fabricated by the NIL technique. The optimized parameters yield narrower dips, as demonstrated through FDTD simulations.

### Preparation and characterization of SLR

**Fig. 2:**
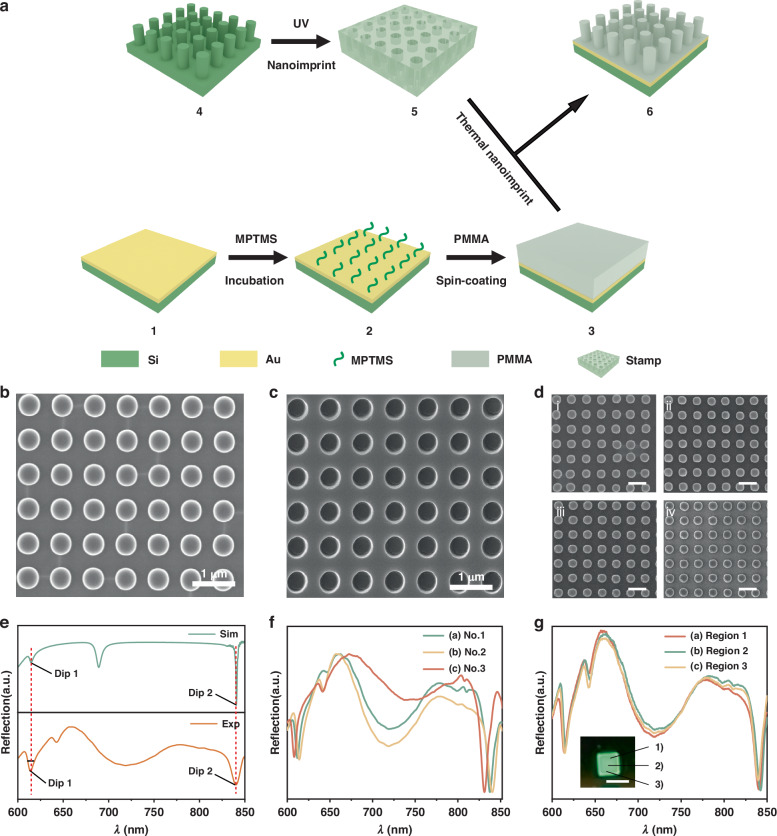
Fabrication and characterization of the SLR array. **a** Schematic diagram illustrating the process of SLR array fabrication: (1) Silicon wafers were coated with gold. (2) A monolayer of MPTMS was self-assembled on gold-coated silicon wafers. (3) A layer of PMMA was spin-coated onto the gold-coated silicon wafers. (4) The silicon mold was previously fabricated by EBL. (5) A stamp was made from the silicon mold by UV nanoimprinting. (6) A SLR array was obtained by thermally nanoimprinting PMMA on the gold-coated silicon wafers. **b** SEM image of the previously fabricated silicon mold. **c** SEM image of the stamp made from the silicon mold. **d** SEM image of the as-prepared SLR array, depicting (i) molding for the first time, (ii) molding for the second time, (iii) molding for the third time, and (iv) molding for the fourth time. **e** Comparison between simulated and experimental reflectance spectra of SLR array. **f** Reflectance spectra of SLR arrays from different arrays of the same batch. **g** Reflectance spectra of different regions with the SLR array. Inset is a photographic image of the SLR array showing the locations for measurement. The effective area of the array is 2 × 2 mm. Scale bars: 1 μm for (**b**–**d**). 2 mm for (**g**)

After optimization of the dimensional parameters on the design of the SLR array, SLR arrays were fabricated through an improved thermal NIL process, as shown in Fig. 2a. The silicon mold was firstly prepared by EBL and reactive ion etching (RIE)^8^. Subsequently, a UV nanoimprint process was employed to create the stamp with complementary structures to the silicon mold^36^. Silicon wafers were used as substrates for the SLR array, where a 10 nm-thick Ti layer and a 100 nm-thick gold layer were deposited on the silicon sequentially using electron beam evaporation. A layer of PMMA resist is spin-coated on the gold surface, and the thickness can be adjusted by varying the concentration of PMMA solution at a specific spin-coating speed. To enhance the adhesion of PMMA on the gold layer, a self-assembled monolayer of (3-mercaptopropyl) trimethoxysilane (MPTMS) was formed on the gold layer before spin-coating. Thereafter, the stamp is placed on PMMA, applying a pressure of 0.2 tons and maintaining a temperature of 135 °C for 5 min. After cooling down to room temperature, the substrate with the nanostructured PMMA layer is separated from the stamp to produce the SLR array. We have fabricated a batch of SLR arrays using the same stamp, and no obvious defects were found on both the stamp and PMMA structures. The fabrication effects of the SLR array are characterized by scanning electron microscopy (SEM). The morphologies of the silicon mold and the stamp are shown in Fig. 2b, c, and the multiple replication results of the SLR arrays are displayed in Fig. 2d, confirming the successful fabrication of the complete arrays.

The reflectance spectra of the fabricated and simulated SLR array were compared in Fig. 2e. The results indicate a satisfying agreement between the simulated and actual SLR array. The FWHM of the fabricated SLR array was 5.1 ± 1.1 nm, which is higher than the theoretical expectation, possibly due to fabrication defects. The *Q* factor of the SLR can be estimated using the formula *Q* = *λ*/Δ*λ*^15^. The *Q* factor value of SLR was up to 154 for wavelength near 615 nm. Additional dips were found in the spectrum of the fabricated SLR array, which could be attributed to the imperfection in the fabrication process.

To assess the consistency of the SLR arrays during successive fabrication processes, spectral measurements were conducted on different batches of arrays, as depicted in Figs. 2f and S5A. The results demonstrate only small variations in the wavelength of the resonance dips among different batches of successively fabricated arrays. To evaluate the uniformity of the arrays, reflectance spectroscopy measurements were performed at three different randomly selected locations on the array, as detailed in Figs. 2g and S4B, which show excellent consistency. These results indicate that the nanoimprint method offers good repeatability and uniformity. Then, we performed a statistical analysis of four slices in the same batch, as shown in Fig. S4. The results indicate that the processing deviation of the same batch is within 10 nm.

These findings affirm the successful fabrication of SLR arrays with excellent uniformity and fabrication repeatability, positioning them as reliable and accurate platforms for various applications. We conducted a comprehensive analysis comparing our fabricating processes with existing SLR fabricating technologies, as shown in Table S1. Moreover, the spectral performance suggests the SLR array holds the potential to achieve high-resolution sensing capabilities.

### The electric field distribution and theoretical RI sensitivity of the array

**Fig. 3:**
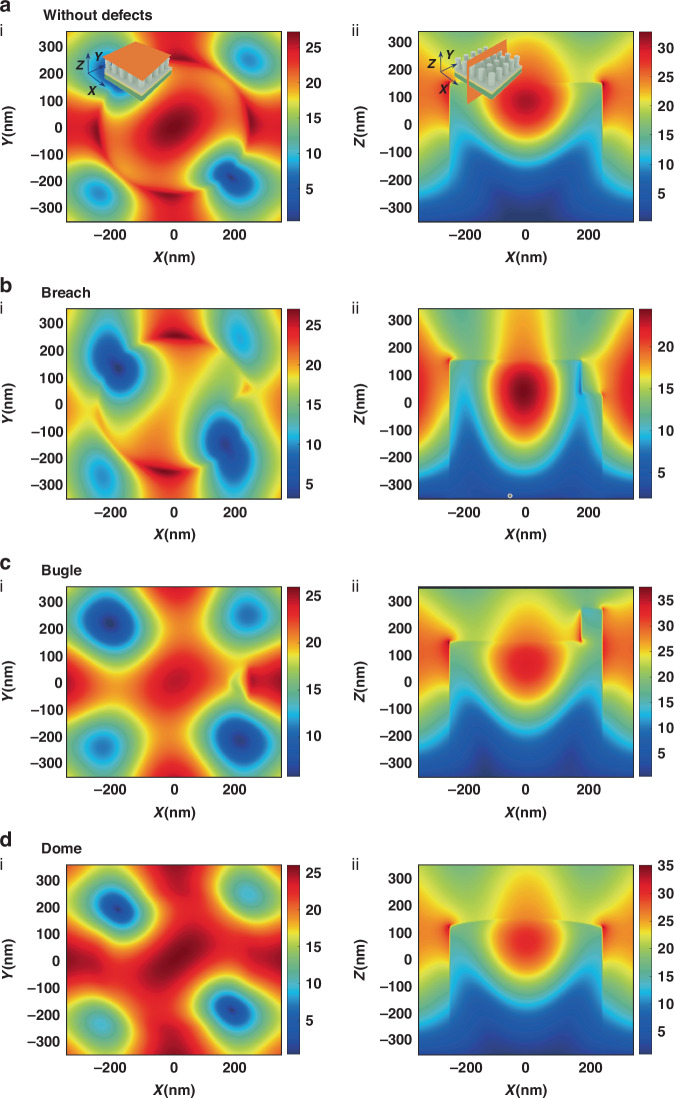
Simulation of electric field distribution on the surface of the SLR array at dip 2. **a** (i) Top view of the electric field distribution on the surface of the array without defects at 10 nm over the pillar. (ii) Side view of the electric field distribution on the surface of the array without defects. **b** (i) Top view of the electric field distribution on the surface of the array with defect of breach at 10 nm over the pillar. (ii) Side view of the electric field distribution on the surface of the array with defect of breach. **c** (i) Top view of the electric field distribution on the surface of the array with defect of bugle at 10 nm over the pillar. (ii) Side view of the electric field distribution on the surface of the array with defect of bugle. **d** (i) Top view of the electric field distribution on the surface of the array with defects of the dome at 10 nm over the pillar. (ii) Side view of the electric field distribution on the surface of the array with defect of dome

To further explore the tolerance of SLR arrays to different preparation defects, we performed simulations to analyze the electric field distribution on the surface of the SLR array with and without defects. Firstly, the electric field distribution on the surface of the SLR array at a wavelength of 841 nm (marked as dip 2 in Fig. 2e) was simulated. Figure 3a demonstrates the top view (i) and side view (ii) of the electric field distribution on the surface of the array without defects. At the wavelength of dip 1, the local electric field is mainly concentrated on the upper surface of PMMA. Figure 3b demonstrates the top view (i) and side view (ii) of the electric field distribution of the array with defect of breach. Figure 3c demonstrates the top view (i) and side view (ii) of the electric field distribution of the array with the defect of the bugle. Figure 3d demonstrated the top view (i) and side view (ii) of the electric field distribution of the array with defects of the dome. The small difference in electric field distributions among the arrays with different types of defects also indicates the high tolerance of the SLR array to defects. The top view and the side view of the electric field distributions on the surface of the array at a wavelength of 615 nm (marked as dip 1 in Fig. 2e) are shown in Fig. S6, which is consistent with that at dip 2.

Furthermore, to evaluate the refractive index sensing performance of the SLR array, we conducted theoretical analyses. We calculated the sensitivity of two SLR dips from the simulated reflectance spectra of the array under environmental refractive index ranging from 1.00 to 1.07 by FDTD. Figure S7A illustrates simulated reflectance spectra of the SLR array in a medium with different refractive indices. The reflectance spectra of dip 1 and dip 2 are shown in Figs. S7B and S7C, respectively. The relationship between the wavelength of the resonance dip and the refractive index is shown in Fig. S7D. By linear fitting, we calculated the sensitivities of the two SLR dips to be 435 nm/RIU and 733 nm/RIU, respectively.

This implies that the resonance dips of the SLR array would shift accordingly to the change in the refractive index of the surrounding medium environment. These findings highlight the potential sensing applications of the SLR array. By monitoring the shifts of the SLR dips, we can infer the variations in the refractive index of the environment near the surface of the SLR array, providing valuable information for the development of sensors based on SLR arrays.

### Enhanced immunoassay using SLR array

To further demonstrate the applicability of the SLR array in immunoassay, the one-step detection of human IgG was performed. The modification process of the anti-IgG onto the surface of the SLR array is illustrated in Fig. S8. Firstly, we modified the surface of the SLR array through esterification with amino^37,38^, then activated the carboxyl groups using NHS/EDC, enabling the connection with anti-IgG. The modification process of the SLR array was characterized by using reflectance spectroscopy and spectral domain phase sensitive interferometry (SD-PSI). The results demonstrated redshifts in the resonance dip during the modification process (Fig. S8B). Figure S8C and S8D show the detailed reflectance spectra of the SLR array during the modification process at around 615 and 841 nm of wavelength, respectively. The redshifts of the resonance dip clearly imply that anti-IgG was successfully modified onto the SLR array. To further confirm the successful attachment of anti-IgG, SD-PSI with high-sensitivity and high dynamic-range phase imaging was performed to monitor the binding process of biomolecules in a microfluidic channel as a cross-validation. As shown in Fig. S8E, an increase in the relative thickness of the surface occurred with the modification of anti-IgG, which also proves the successful modification of anti-IgG on the surface.

**Fig. 4:**
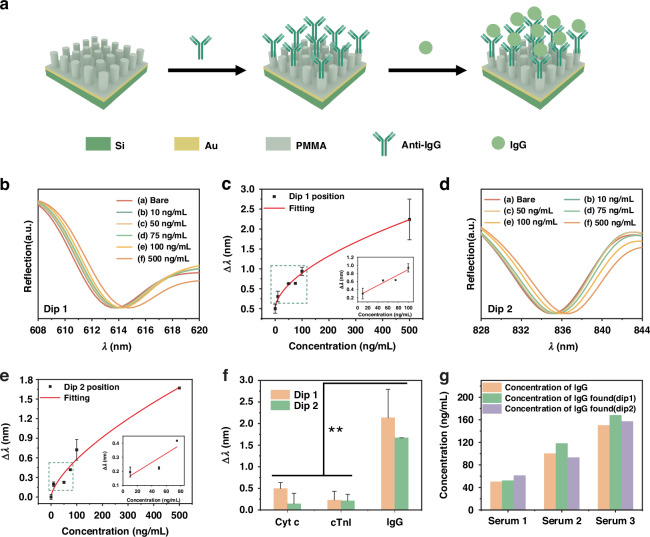
IgG detection using the SLR array. **a** Diagram illustrating the modification of the SLR array for immunoassay. Anti-IgG antibody was modified on the SLR array, and then IgG (antigen) was detected by the antibody-modified SLR array. **b** Spectra of the array at dip 1 in different concentrations of IgG. **c** The fitting of spectral shift (Δ*λ*) of dip 1 in (**b**), where the inset shows the linear fit of the linear part in the fitted curve. **d** Spectra of the array at dip 2 in different concentrations of IgG. **e** The fitting of spectral shift (Δ*λ*) of dip 2 in (**d**), where the inset is the linear fitting of the linear part of the fitting curve. **f** Comparison of spectral shift induced by IgG, Cyt c, and cTnl at a concentration of 500 ng/mL. **P < 0.01. **g** Application of the SLR array for the detection of serum samples with different concentrations of IgG. Error bars represent the standard deviation of three replicate measurements (*n* = 3)

Then, the biosensing performance of the SLR array was evaluated using different concentrations of IgG as target molecules, as shown in Fig. 4a. Figure 4b reveals a redshift in the resonance dip with increasing IgG concentration. Figure 4c shows the spectral shift of dip 1 against IgG concentration. The red shift of the dip ranges from average 0.3 to average 2.2 nm as IgG concentration increased from 10 to 500 ng/mL, and the modified SLR array exhibited a limit of detection (LOD) of 12.44 ng/mL. The inset shows the linear fit of the linear part in the fitted curve. Figure 4d reveals the redshift of dip 2 as the IgG concentration increases. As shown in Fig. 4e, the modified SLR array exhibited a low LOD of 609 pg/mL (i.e. 3.9 pM), and the linear range is 10–100 ng/mL. It is worth mentioning that the LOD of dip 2 is lower than dip 1. These results demonstrate the array’s high sensitivity in sensing human IgG molecules, enabling effective immunoassay. The LSR sensor is suitable for low-concentration immunoassay.

Furthermore, to assess the selectivity of the SLR array sensor, we also measured cytochrome c (Cyt c) and cardiac troponin I (cTnI) under the same condition, respectively. As shown in Fig. 4f, the capture of IgG makes a remarkable red-shift, which is much larger than that induced by Cyt c and cTnI. The results confirm the selectivity of the SLR array sensor for IgG detection. We further applied the SLR array sensor to the practical detection of IgG in spiked serum samples. IgG was added to artificial serum at known concentrations of 50 ng/mL, 100 mg/mL, and 150 ng/mL, respectively. Specific parameters are shown in Table S2. Figure 4g shows the ability of the array to recover different concentrations of IgG from three serum levels. The concentration recovered from dip 1 and dip 2 is close to the actual concentration, reaching a consistence of 99%, which suggests the potential applicability of the SLR array for clinical detection of IgG.

Overall, these findings highlight the promising potential of the SLR array for immunoassay applications. The successful surface modifications and the low LOD achieved underscore the array’s sensitivity and suitability for precise and accurate detection of target analyses, such as IgG.

Immunoassays are essential for detecting physiologically relevant protein indicators in clinical diagnostics^39^. Traditional immunoassay methods, including enzyme-linked immunosorbent assay, fluorescence immunoassay, and chemiluminescence, are often associated with complicated procedures, high reagent costs, and extended assay times. Our defect-insensitive cylindrical SLR array is designed to address these challenges by providing enhanced sensitivity and specificity in immunoassay applications. The SLR array achieves high refractive index sensitivity due to its narrow FWHM and sharp resonance peaks, which are critical for detecting subtle changes associated with antigen-antibody interactions. To demonstrate the array’s potential in enhanced immunoassay, we conducted both theoretical analyses and experimental tests that illustrate its capability to match and potentially exceed the sensitivity levels of commercial immunoassays^40^. Our results indicate that the SLR array can detect biomolecular binding events with high precision and minimal noise, even in complex biological matrices.

## Conclusion

In conclusion, we have designed a novel SLR array with excellent tolerance for fabrication defects and nanoscale replication accuracy, which demonstrates great potential in the field of immunoassay. Specifically, the array boasts a three-layer cylindrical structure and was fabricated through NIL. This innovative structure design significantly improves the batch-to-batch consistency of the SLR array. By integrating metal evaporation and NIL techniques, we have efficiently fabricated the SLR array in large quantities. Despite the simplicity of SLR preparation steps, the array retains the desirable characteristics of narrow spectral dips, with a remarkable FWHM as narrow as 5.1 nm. Through the modification of PMMA with hexamethylene diamine, the array exhibits the capability to immobilize anti-IgG for the one-step detection of IgG with a linear relationship within the concentration range of 2–150 ng/mL. The sensitivity of the array for IgG detection achieves an exceptional detection limit as low as 609 pg/mL. The successful development and characterization of the novel SLR array highlight its significant contributions to the SLR field of practical application. The array’s scalability, reproducibility, and high sensitivity make it a promising platform for a wide range of sensing and detection applications beyond immunoassay.

## Experimental section

### Materials

N-hydroxysuccinimide (NHS) and N-ethyl-N’(3-dimethylaminopropyl) carbodiimide hydrochloride (EDC-HCl) were supplied by Aladdin Co. Ltd. (China). Single-side polished silicon wafers were purchased from Ruigeruisi (China), Phosphate-buffered saline (PBS; pH 7.4) was supplied by Gbico (UK), Isopropyl alcohol and glycerol was purchased from Fuyu chemical reagent (China). Ethanol was purchased from an Energy chemical reagent (China). Ormostamp was supplied by Micro Resist Technology (Germany). PMMA was purchased from Chuangsuhua Co. Ltd. (China). (3-methacryloxypropyl) trimethoxysilane (MPTMS) and Anisole, 1H,1H,2H,2H-perfluorocranyl trichlorosilane (FDTS), 6-hexamethtlenediamine were purchased from Macklin reagent (China). Deionized water was prepared by a Milli-Q Advantage A10 water system (Millipore, Billerica, MA, USA) with a resistivity of 18.2 MΩ cm. All chemicals and solvents used were of analytical grade and were used as received.

### Fabrication of SLR array

The template of the array was created using EBL lithography. Subsequently, the stamp was generated through UV nanoimprinting. Ormostamp was added dropwise to the array template and covered with glass. Following a fifteen-minute interval to allow for the leveling of the ormostamp, the samples underwent UV light curing (Uvitron, USA) for 300 s to ensure the complete cross-linking of the photoresist. Subsequently, the samples were placed on a 150 °C hot plate for 30 min for effective hard baking. After these steps, the ormostamp templates were placed in a vacuum chamber at 95 °C and evacuated with FDTS for 15 min to form an anti-adhesive monomolecular layer on the template’s surface. Next, a silicon wafer was evaporated with a 100-nm thickness of gold by electron beam evaporation (Wavetest, China). To enhance the adhesion of the gold surface, the gold-coated silicon wafer was immersed in 25 mM of MPTMS solution for 1 h, so that MPTMS on the gold surface was self-assembled on the gold surface for linking the gold layer with PMMA. Subsequently, a layer of 8% PMMA was spin-coated onto the silicon substrate at 4000 rpm and baked at 140 °C for 30 min before use. The stamp was placed above it and inserted into the T-NIL system (Specac, UK). A force of 0.2 tons and a temperature of 135 °C for 5 min were applied. The counter nanostructures of the ormostamp template pattern were imprinted into the PMMA. This process allows for the preparation of the SLR array. Since the template is reusable during the nanoimprinting process, SLR arrays can be fabricated in bulk.

### Optical characterizations of SLR array

The reflectance spectra of the SLR array sensor were obtained using a spectrometer (HRS-300, Princeton Instruments). The resolution of the instrument is 0.04 nm. All reflectance spectra are normalized to the reflectance spectrum of a gold reflector as a reference. The morphology of the SLR array was characterized using an Auriga scanning electron microscope (Zeiss, Germany) operating at a voltage of 50 kV.

### Numerical simulations

The simulation of the reflectance spectrum and electric field intensity distribution of the SLR array was performed by a commercial finite-difference time-domain software (FDTD Solutions, Lumerical Inc.). Periodic boundary conditions and perfectly matched layers were employed for the horizontal (*x*- and *y*-) and vertical (*z*-) directions in the simulation, respectively. On the top of the substrate and Au layer, a PMMA layer was added, whose refractive index is 1.492. The dielectric constants of Au were taken from Palik. The simulations of the scattering efficiencies were done using a flat light source with a broadband (500–900 nm) beam incident from the air and along the normal direction.

### Immobilization of goat anti-human IgG onto SLR array

The SLR array was immersed in 25 mM 6-hexamethylenediamine for 1 h to form a self-assembled monolayer of amine. Soon after, the array was rinsed with ethanol and then incubated in 1:4 aqueous solutions of 50 mM of N-hydroxysuccinimide (NHS) and 200 mM of N-ethyl-N-(3-diethylaminopropyl) carbodiimide (EDC) for 2 h. Then, the surface that had been modified was immersed in a 2 μg/mL goat anti-human IgG in 0.01 M phosphate-buffered saline (PBS) buffer solution for 1 h to complete the immobilization process.

### Biomolecular detection using SLR array

Goat anti-human IgG-modified SLR arrays were placed on a microspectrometer. Then 1 mL human IgG (Thermo, USA) with concentrations ranging from 10 to 500 ng/mL in 0.01 M PBS buffer solution was loaded into the cuvette and incubated for 30 min before collection of reflectance spectra.

### Statistical analysis

Unless otherwise stated, data were expressed as mean ± standard deviation. Statistical comparisons were made with Student’s *t*-test in SPSS 22 software. For the Student’s *t*-test, **p < 0.01 was considered statistically very significant.

## Supplementary information

Defect-insensitive cylindrical surface lattice resonance array and its batch replication for enhanced immunoassay
