# Supplementary material for: Defect-insensitive cylindrical surface lattice resonance array and its batch replication for enhanced immunoassay
Source: Microsyst Nanoeng. 2024 Nov 13;10:168. doi: 10.1038/s41378-024-00793-3 (PMC11560952; doi:10.1038/s41378-024-00793-3)
Supplement: Supplementary file 1 — Defect-insensitive cylindrical surface lattice resonance array and its batch replication for enhanced immunoassay [file 41378_2024_793_MOESM1_ESM.pdf]

Supporting Information for:

Defect-insensitive cylindrical surface lattice resonance array and its batch replication for enhanced immunoassay

Bin Zhou†, Chao Hu†, Haoyang Li, Xiangyi Ye, Baohua Wen, Zhangkai Zhou, Jingxuan Cai*, and Jianhua Zhou*

†These authors contributed equally to the work.

Dr. B. Zhou, C. Hu, B. H. Wen, Dr. J. X. Cai, Prof. J. H. Zhou

1 Key Laboratory of Sensing Technology and Biomedical Instruments of Guangdong Province, School of Biomedical Engineering, Shenzhen Campus of Sun Yat-sen University, Shenzhen 518107, China.

H.Y. Li, Prof. Z. K. Zhou

School of Physics, State Key Laboratory of Optoelectronic Materials and Technologies, Sun Yat-sen University, Guangzhou 510275, China

E-mail: zhoujh33@mail.sysu.edu.cn (J. H. Zhou); caijx27@mail.sysu.edu.cn (J. X. Cai)

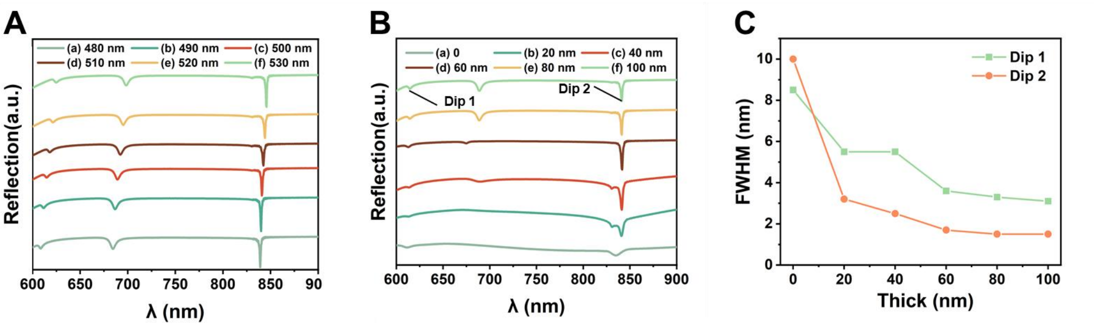

Supplementary Figure S1. (A) Effect of pillar diameters on spectra. (B) Effect of the gold layer thickness on spectra. (C) Effect of the gold layer thickness on the full width at half maximum (FWHM) of dip 1 and dip 2.

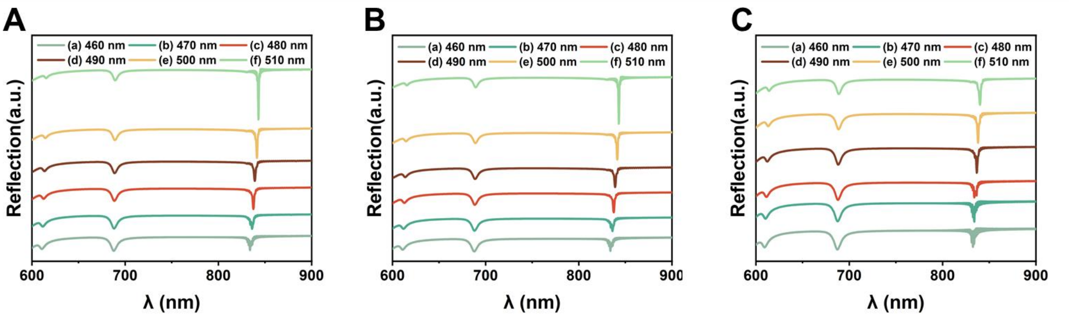

Supplementary Figure S2. Reflectance spectra of SLR array with defects of breach in different heights from 460 – 510 nm. (C) Reflectance spectra of SLR array with defects of bugle in different heights from 460 – 510 nm. (D) Reflectance spectra of SLR array with defects of dome in different heights from 460 – 510 nm.

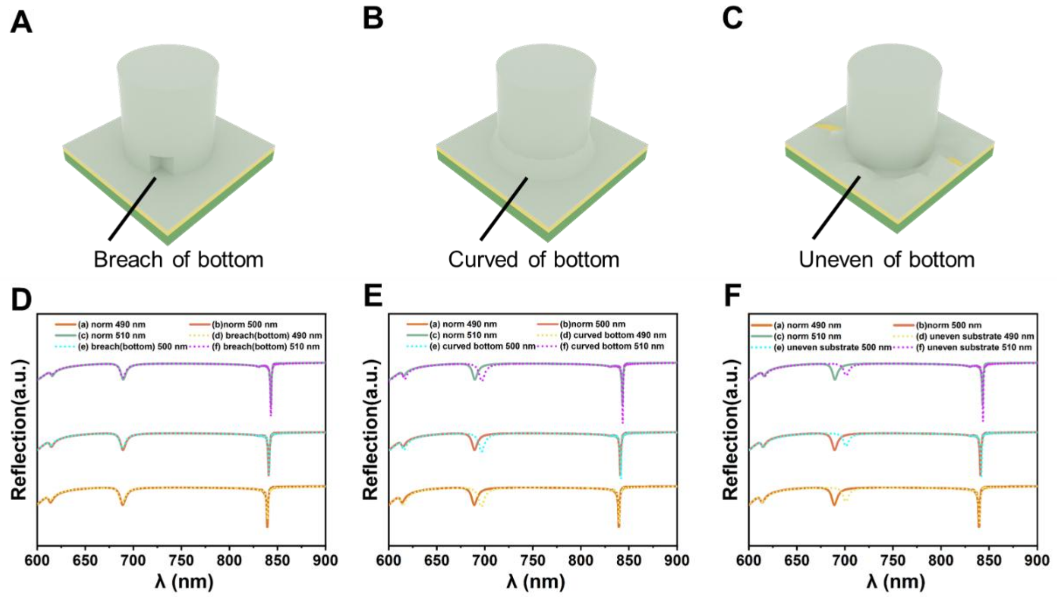

Supplementary Figure S3. Simulation of reflectance spectra of SLR array with different types of structural defects: breach of bottom, curved of bottom and uneven of bottom. (A) Schematic diagram showing the structure defect of breach of bottom. (B) Schematic diagram showing the structure defect of curved of bottom. (C) Schematic diagram showing the structure defect of uneven of bottom. (D) Reflectance spectra of the SLR array with defects of breach compared to the SLR array without defects. (E) Reflectance spectra of the SLR array with defects of curved of bottom compared to the SLR array without defects. (F) Reflectance spectra of the SLR array with defects of uneven of bottom compared to the SLR array without defects.

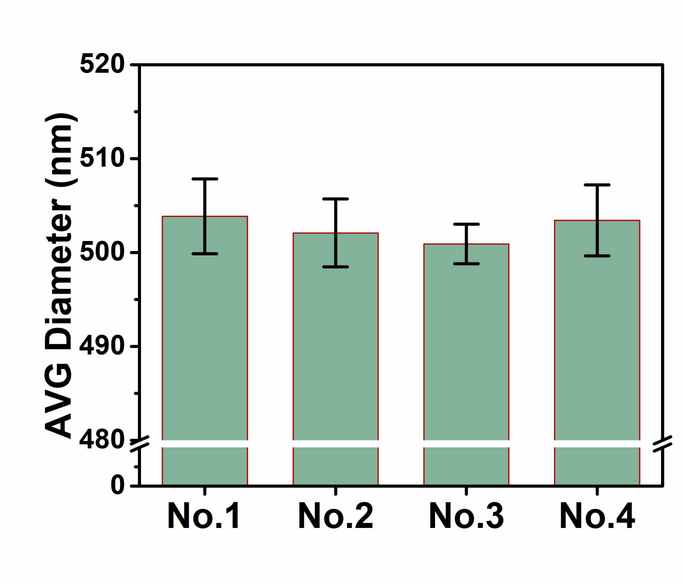

Supplementary Figure S4. Statistical analysis of nine pillars of the four slices in the same batch. It indicates that the fabrication processing deviation of the same batch is within 10 nm.

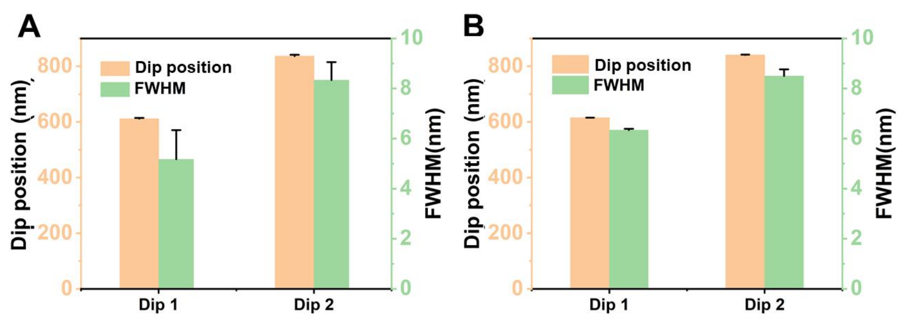

Supplementary Figure S5. (A) Statistic of dip position and FWHM of reflectance spectra in different batches of the SLR array, indicating the spectra consistency of the SLR array fabricated in different batches. (B) Statistic of dip position and FWHM of reflectance spectra in different regions of the SLR array, indicating the consistency of the flatness of different samples fabricated by nanoimprinting.

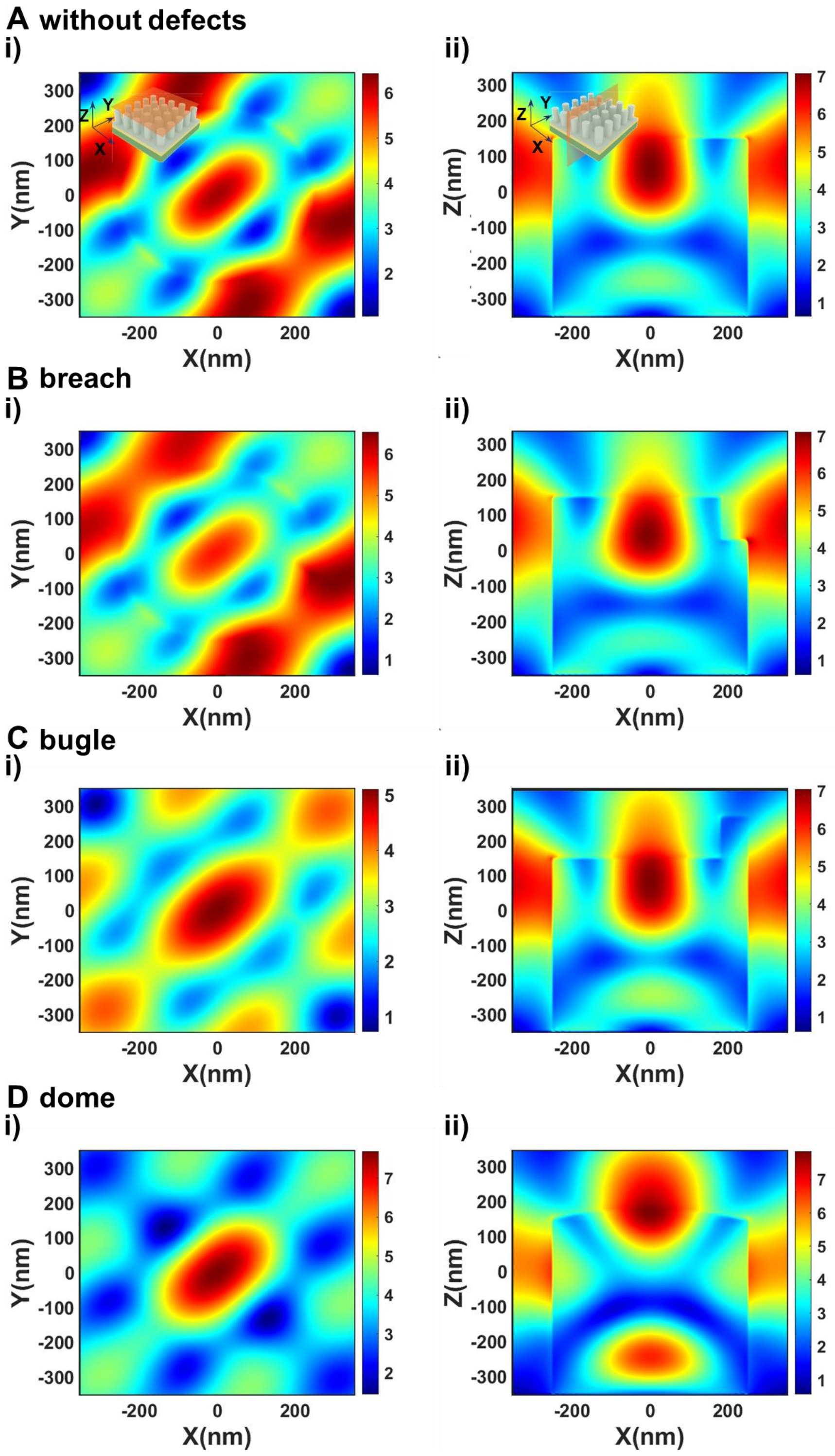

Supplementary Figure S6. Simulation of electric field distribution on the surface of the SLR array at dip 1. (A) i) The top view of the electric field distribution on the surface of the array without defects at 10 nm over the pillar. ii) Side view of the electric

field distribution on the surface of the array without defects. (B) i) The top view of the electric field distribution on the surface of the array with defect of breach at 10 nm over the pillar. ii) Side view of the electric field distribution on the surface of the array with defect of breach. (C) i) The top view of the electric field distribution on the surface of the array with defect of bugle at 10 nm over the pillar. ii) Side view of the electric field distribution on the surface of the array with defect of bugle. (D) i) The top view of the electric field distribution on the surface of the array with defects of dome at 10 nm over the pillar. ii) Side view of the electric field distribution on the surface of the array with defect of dome.

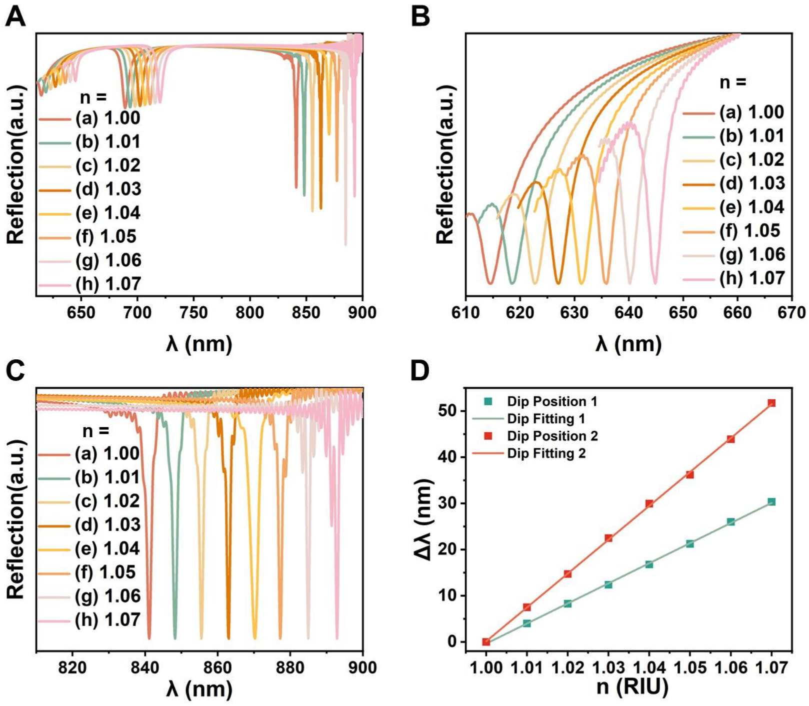

Supplementary Figure S7. Theoretical RI sensitivity of the SLR array. (A) Spectra of the array in different refractive index (n) environments. (B) Spectra of the array at dip 1 in different refractive index environments. (C) Spectra of the array at dip 2 in different refractive index environments. (D) Dip shifts of dip 1 and dip 2 in different refractive index environments. Dip 1: $\Delta\lambda = 435n - 435$; Dip 2: $\Delta\lambda = 733n - 733$. $R^2 = 0.99$.

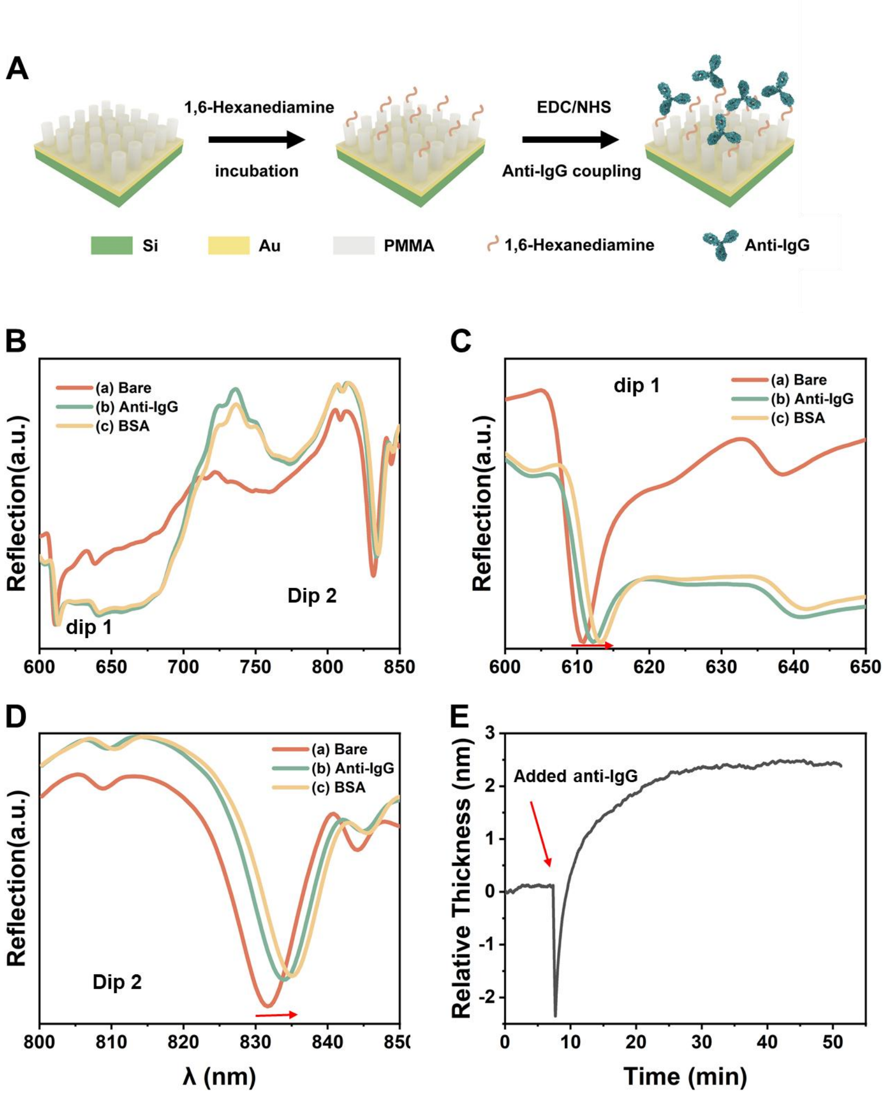

Supplementary Figure S8. (A) Reflectance spectra of the SLR array during modification of anti-IgG. (B) Spectral shift of the SLR array at dip 1 during modification of anti-IgG. (C) Spectral shift of the SLR array at dip 2 during modification of anti-IgG. (D) Measuring the anti-IgG modification process of the SLR array by spectral domain phase sensitive interferometry.

Table S1. Comparison of the fabrication technique of SLR.

Fabricated Method	Cost	Spectral sensitivity	Experimental FWHM	Reproducibility.	References
Electron-beam lithography	High	High	~ 50 nm	Low	*Nanophotonics*, **2023**, 12: 3721-7
Electron-beam lithography	High	High	32 nm	Low	*Nat. Commun.*, **2022**, 13: 5737.
Colloidal lithography	Low	—	~100 nm	Low	*Adv. Mater.* **2020,** *32*: 2001330.
Colloidal lithography	Low	—	2.7 ~ 3.2 nm	Low	*ACS. Appl. Mater. Interfaces,* **2024**, 16: 1259-67.
Colloidal lithography	Low	—	4 nm	Low	*ACS Nano,* **2023**, 1: 725-34.
Soft lithography	Low	—	~ 9 nm	Low	*ACS Nano*, **2019**, 13: 9038-47.
**Nanoimprint lithography**	**Low**	**High**	**5.1 ± 0.1 nm**	**High**	**Our work**

"—" had no related report

Table S2. The application of the SLR array sensor for the detection of serum samples spiked with different concentrations of IgG.

**Sample**	**Concentration of IgG**	**Concentration of IgG found at dip 1**	**Recovery at dip 1**	**Concentration of IgG found at dip 2**	**Recovery at dip 2**
serum 1	50 ng/mL	52 ng/mL	104 %	61 ng/mL	122 %
serum 2	100 ng/mL	118 ng/mL	118 %	93 ng/mL	93 %

serum 3	150 ng/mL	168 ng/mL	112 %	157 ng/mL	104 %
